# Genetics, pharmacotherapy, and dietary interventions in childhood obesity

**DOI:** 10.3389/jpps.2024.12861

**Published:** 2024-05-28

**Authors:** Joe Eun Son

**Affiliations:** School of Food Science and Biotechnology, Research Institute of Tailored Food Technology, Kyungpook National University, Daegu, Republic of Korea

**Keywords:** childhood obesity, genetics, pharmacotherapy, dietary intervention, personalized therapy

## Abstract

Childhood obesity has emerged as a major global health issue, contributing to the increased prevalence of chronic conditions and adversely affecting the quality of life and future prospects of affected individuals, thereby presenting a substantial societal challenge. This complex condition, influenced by the interplay of genetic predispositions and environmental factors, is characterized by excessive energy intake due to uncontrolled appetite regulation and a Westernized diet. Managing obesity in childhood requires specific considerations compared with adulthood, given the vulnerability of the critical juvenile–adolescent period to toxicity and developmental defects. Consequently, common treatment options for adult obesity may not directly apply to younger populations. Therefore, research on childhood obesity has focused on genetic defects in regulating energy intake, alongside pharmacotherapy and dietary interventions as management approaches, with an emphasis on safety concerns. This review aims to summarize canonical knowledge and recent findings on genetic factors contributing to childhood obesity. Additionally, it assesses the efficacy and safety of existing pharmacotherapies and dietary interventions and suggests future research directions. By providing a comprehensive understanding of the complex dynamics of childhood obesity, this review aims to offer insights into more targeted and effective strategies for addressing this condition, including personalized healthcare solutions.

## Introduction

Childhood obesity has emerged as a critical global health concern, notably in developed countries where obesity rates among children and adolescents have nearly tripled in the last 30 years. Projections by the World Obesity Federation anticipate that by 2030, approximately 250 million children worldwide will be obese [1–3]. This condition markedly increases the risk of chronic diseases, including fatty liver and type 2 diabetes [1, 4–8]. The persistent transition from obesity in childhood to adulthood is especially concerning, with >80% of affected adolescents expected to remain obese as adults [9]. Beyond impacting physical health, this trend affects self-esteem, social relationships, and future economic prospects, underscoring the urgent need for action [1, 10, 11].

The etiology of childhood obesity is multifaceted, involving a complex interplay of genetic and environmental factors [1]. Advances in genetic research have illuminated the role of specific genetic factors influencing energy homeostasis, particularly appetite regulation [12, 13]. Studies on the genetics of obesity have identified key genes as pivotal contributors to the condition, enhancing our understanding of its biological mechanisms and opening new avenues for preventive and therapeutic strategies.

In addition to deepening our understanding of the mechanisms involved in obesity, genetic insights also drive the development of pharmacotherapies targeting specific metabolic pathways. Such treatments have shown promise in adults, signaling a potential shift toward more effective obesity management. However, their use in children and adolescents remains limited, being primarily reserved for cases of severe obesity and diabetes in adolescents, where treatment benefits are considered to outweigh the risks [14]. Alongside this cautious approach, new medications are under development, emphasizing improved efficacy and safety, accompanied by more rigorous clinical validation.

Environmental factors, particularly diet and lifestyle with reduced physical activity, also play a crucial role in childhood obesity development [15]. Modern dietary patterns, often labeled the Western diet, are mainly characterized by a high caloric intake of saturated fats and refined carbohydrates and frequent consumption of sugar-sweetened beverages, which closely correlate with rising childhood obesity rates [16–20]. Consequently, management strategies for childhood obesity are increasingly focused on dietary interventions, such as ketogenic diets, fasting-based interventions, and dietary supplements. Ongoing research explores the effectiveness and safety of these dietary interventions in preventing and treating childhood obesity.

**FIGURE 1:**
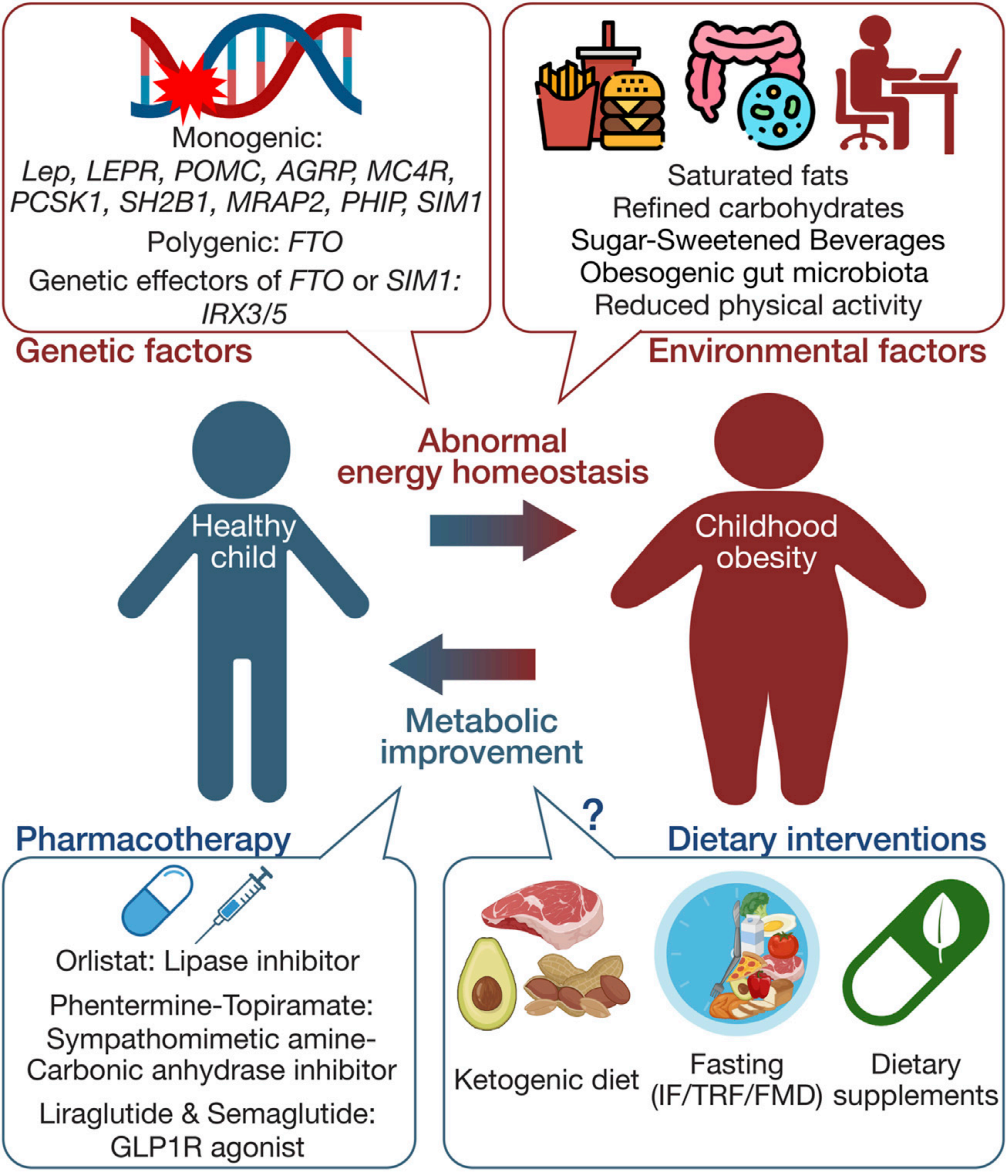
Schematic summary of the multifaceted etiology and management approaches in childhood obesity. This figure illustrates the complex interplay between genetic and environmental factors influencing the development of childhood obesity. It also delineates primary management strategies, including pharmacotherapy and dietary interventions. The schematic was created using illustrations from https://biorender.com.

This review aims to summarize current knowledge on genetic factors contributing to childhood obesity, evaluate the efficacy and safety of existing pharmacotherapies and dietary interventions (outlined in Figure 1), and suggest directions for future research. By presenting a comprehensive understanding of the complex dynamics involved in childhood obesity, this review highlights potential approaches for more effective and safe treatment strategies, ultimately providing foundations for tailored interventions addressing genetic predispositions and environmental influences.

## Genetic factors in childhood obesity

Twin studies have highlighted the heritability of obesity, with estimates for body mass index (BMI) heritability reaching up to 70% [21, 22]. The genetic landscape of childhood obesity has been extensively explored, revealing multiple genetic factors contributing to its development (Figure 1). Childhood obesity is predominantly polygenic, involving multiple genes, each contributing modestly but collectively exerting a substantial impact [23, 24]. In contrast to monogenic forms of obesity, resulting from single genetic defects with pivotal effects [25], the genetic predisposition to childhood obesity in the broader population is shaped by numerous common genetic variants, collectively exerting a substantial impact on the obesity phenotype.

The advent of genome-wide association studies (GWAS) has markedly advanced our understanding of the genetic basis of obesity. A landmark discovery involved identifying variants in the fat mass and obesity-associated (*FTO*) gene as a major risk factor for obesity in the general population and severe childhood obesity. The strongest association was noted for single-nucleotide polymorphisms in the first intron of *FTO*. The influence of *FTO* gene variants on energy homeostasis is mediated through their impact on appetite regulation, with certain variants linked to increased energy intake and high-calorie food preference [26–29].

Monogenic obesity, although rare, is predominantly identified in patient cohorts with severe and early-onset obesity, highlighting its strong correlation with severe childhood obesity. Monogenic obesity is mainly attributed to genetic mutations associated with the central regulation of energy homeostasis, particularly appetite control driven by the leptin–melanocortin signaling pathway [21]. Genes implicated in monogenic obesity include *Lep* (*leptin*), *LEPR* (*leptin receptor*), *POMC* (*pro-opiomelanocortin*), *AGRP* (*agouti-related protein*), *MC4R* (*melanocortin 4 receptor*), *PCSK1* (*proprotein convertase subtilisin/kexin type 1*), *SH2B1* (*SH2B adaptor protein 1*), *PHIP* (*proline-rich protein 5*), *MRAP2* (*melanocortin 2 receptor accessory protein 2*), and *SIM1* (*single-minded 1*) [30–42]. In most monogenic obesity cases, genetic mutations drive abnormal feeding behavior, resulting in early-onset, severe hyperphagic obesity.

Recently, the *Iroquois* homeodomain transcription factor genes *IRX3* and *IRX5* have emerged as novel genetic determinants in human obesity, revealing the complex genetic interactions underlying this condition. Known for their similar expression patterns and cooperative roles during mammalian development, the *IRX3* and *IRX5* genes have been implicated in obesity through interactions with intronic *FTO* locus variants. Chromatin conformation capture techniques revealed that these variants physically interact with *IRX3* and *IRX5* promoter regions, serving as enhancers that increase *IRX3*/*IRX5* expression levels in the hypothalamus and adipose tissue [43–45]. This upregulation influences crucial physiological processes, including feeding control, thermogenesis, and adipogenesis, positioning *IRX3*/*IRX5* as central mediators of *FTO* variant–associated obesity effects [46–48]. Notably, *Sim1* interacts with *IRX3*/*IRX5*. Specifically, loss-of-function mutations in *SIM1* are linked to hyperphagic childhood obesity, and *Sim1* haploinsufficiency leads to ectopic expression of *IRX3*/*IRX5* in the hypothalamus in mice, causing neurodevelopmental defects and contributing to appetite dysregulation and hyperphagic obesity [49]. Further research is warranted to explore the mechanistic evidence for the tissue- or cell-type-specific roles of *IRX3*/*IRX5*, particularly their involvement in regulating energy homeostasis. This evolving genetic narrative emphasizes the need to elucidate these pathways for further advancements in childhood obesity prevention and treatment.

## Pharmacotherapy in childhood obesity

Managing childhood obesity often involves pharmacological intervention, especially in cases where a child presents with a severe obesity phenotype and critical health issues. The cautious application of pharmacotherapy in young patients with obesity stems from concerns regarding potential long-term impacts on growth and overall development. Current pharmacotherapy options are predominantly limited to adolescents, particularly in cases of severe obesity with accompanying comorbid conditions. Pharmacotherapies currently used in childhood obesity cases are summarized in Figure 1.

### Classical pharmacotherapy: orlistat and phentermine

Among the drugs approved for adults, only a few have received approval for childhood–adolescent obesity treatment. Until the early 2020s, orlistat and phentermine were the sole U.S. Food and Drug Administration (FDA)-approved medications for this purpose [50, 51]. Orlistat, a lipase inhibitor, reduces the hydrolysis of ingested triglycerides, decreasing gastrointestinal fat absorption. Clinical trials have demonstrated its efficacy in BMI reduction compared with placebo groups [52], leading to its approval for use in adolescents aged ≥12; however, orlistat has potential side effects, including diarrhea and hepatic injury, resulting in dropout rates of around 35%–75% within 3 months [52–54]. Long-term use of orlistat may disrupt the absorption of fat-soluble vitamins and minerals, negatively impacting growth or pubertal development [55, 56].

Phentermine, a sympathomimetic amine anorectic, is FDA-approved for monotherapy in adolescents aged ≥16 with severe obesity and additional related health complications. A recent clinical advancement involved the FDA approving the phentermine–topiramate combination for weight loss in obese individuals aged ≥12. Topiramate, originally an antiepileptic agent, contributes to weight loss by inhibiting carbonic anhydrase and increasing γ-aminobutyric acid (GABA) activity, suppressing appetite [57–59]. This combination, leveraging distinct mechanisms, offers a more effective weight loss solution than either drug alone, allowing for lower doses of each medication and enhancing overall treatment efficacy and safety profile. Phentermine/topiramate may pose safety concerns such as mood disorders, cognitive impairment, nephrolithiasis, cardiac risks, and teratogenic effects [60].

Setmelanotide, a melanocortin-4 receptor (MC4R) agonist approved by the FDA in 2020, offers a targeted pharmacological approach for managing monogenic obesity linked specifically to *POMC*, *PCSK1*, or *LEPR* genetic deficiencies [61, 62]. These genetic variants can disrupt signaling through the MC4R pathway, leading to hyperphagia and severe early-onset obesity [21, 63]. MC4R agonist serves as an alternative activator of the MC4R pathway in patients who have POMC deficiencies due to mutations in either *POMC* or *PCSK1* and in those with *LEPR* deficiencies caused by mutations in *LEPR*, which is crucial for POMC function. Hence, the MC4R agonist effectively reduces hyperphagia and promotes weight loss for treating severe obesity linked to these specific genetic disorders [62, 64, 65]. While its effectiveness in clinical trials is notable, its application is limited to these particular genetic disorders and not applicable to general childhood obesity. Setmelanotide therapy is associated with potential side effects, including skin hyperpigmentation, sexual dysfunction, depression, and suicidal ideation [66].

### Innovative pharmacotherapy: GLP-1 receptor agonists

Glucagon-like peptide-1 receptor agonists (GLP1RAs), such as liraglutide and semaglutide, have become pivotal pharmacological agents for managing obesity. Originally developed to treat type 2 diabetes, GLP1RAs unexpectedly induce weight loss. Studies have indicated that GLP1RAs primarily act on the central nervous system to reduce appetite, delay gastric emptying to prolong satiety and alter brain pathways that decrease reward-driven eating behaviors. Ultimately, these actions lead to decreased energy intake and promote weight loss in general obesity and syndromic monogenic forms of obesity, including Prader-Willi syndrome and MC4R mutations [67–74]. Having successfully promoted weight loss in adults, GLP1RAs have received FDA approval, which was extended to adolescents. Specifically, liraglutide treatment has resulted in notable BMI reductions without negative impacts on pubertal development or growth, making it an appropriate option for adolescents aged ≥12 [75, 76]. Recent preliminary investigations into the safety and effectiveness of liraglutide in the 6–12 age group resulted in the initiation of the SCALE KIDS clinical trial, a study assessing its viability as a childhood anti-obesity treatment [77]. Additionally, semaglutide also received FDA approval for weight management in adolescents aged ≥12 with severe obesity in 2022 [78, 79]. This represents a major advancement in expanding therapeutic options for childhood obesity management.

Tirzepatide, recently approved for chronic weight management in adults, is a dual agonist targeting GLP1R and glucose-dependent insulinotropic polypeptide receptor (GIPR), offering a novel approach to obesity treatment by simultaneously enhancing glucose regulation and reducing appetite [80, 81]. It has been shown that GLP1R–GIPR dual agonist is superior in weight reduction to GLP1RAs and offers additional benefits, including improved insulin sensitivity, lipid profiles, and blood pressure [82, 83]. This demonstrates groundbreaking potential in the pharmacology of obesity and related metabolic diseases. Building on its success in adults, tirzepatide is currently in phase 1 clinical trials for children and adolescents aged 6–11 and 12–17 to assess its safety and efficacy. This expansion into pediatric studies reflects a proactive step towards addressing childhood obesity, providing hope for a new, effective treatment option that could mitigate the long-term health consequences associated with early-onset obesity. In the future, the development of novel and effective drugs with favorable safety profiles is expected to revolutionize the approach to treating childhood obesity treatment, even within younger populations. Potential side effects of GLP1RAs or GLP1R–GIPR dual agonists include gastrointestinal symptoms, such as nausea, vomiting, diarrhea, cardiovascular conduction abnormalities, and sinus tachycardia [84, 85].

### Off-label medications

Off-label medication refers to the use of pharmaceutical drugs for an unapproved age group, dosage, or condition. In the context of childhood obesity, metformin is a common example of an off-label medication. Metformin is a well-established, approved option for managing type 2 diabetes in adults and adolescents [86, 87]. Some research suggests that metformin may be effective for weight loss [88–91]; however, due to its modest and inconsistent weight-loss effects, the FDA has yet to approve metformin as a weight-loss agent. Consequently, its use in treating obesity in children has also not received official approval. Nonetheless, multiple lines of evidence demonstrate metformin’s favorable effects on weight management in children and adolescents with obesity, along with a safe profile. This makes metformin a viable and accessible option for off-label use in combating childhood obesity [92, 93]. Although metformin’s efficacy and safety profile are established for children, its off-label use still introduces potential risks. The lack of comprehensive clinical data specifically for treating childhood obesity means that the potential benefits must be cautiously weighed against risks that are not fully understood or might be underestimated. Consequently, the use of off-label medications such as metformin in treating childhood obesity requires careful consideration and underscores the necessity for more rigorous research to confirm their safety and effectiveness in these younger patients.

## Dietary interventions in childhood obesity

Dietary interventions play a pivotal role as an alternative strategy for addressing childhood obesity, particularly as pharmacotherapy is often reserved for severe cases accompanied by additional metabolic complications [94]. These interventions, focusing on altering dietary habits and behaviors, aim to cultivate healthy eating practices conducive to long-term weight management and overall health enhancement. Emerging dietary strategies, including ketogenic diets, fasting-based interventions, and dietary supplements, are gaining attention for their potential in combating childhood obesity (Figure 1).

### Ketogenic diet

The ketogenic diet, characterized by high fat and low carbohydrate intake, prompts the body to convert fats and ketone bodies for energy, entering ketosis [95]. This metabolic shift makes the diet a popular non-pharmacological option for obesity management, given its potential to promote weight loss through enhanced lipolysis and reduced insulin levels [96–98]. Although the ketogenic diet is considered beneficial for obesity-related metabolic and cardiovascular risk factors in adults [99, 100], its role in childhood weight management is still being explored. Clinical trials and animal studies have shown the diet’s effectiveness in promoting weight loss and addressing metabolic issues caused by obesity [101]. However, the long-term safety and efficacy of ketogenic diets in the pediatric population require further investigation. Specifically, maintaining a ketogenic diet for extended periods may lead to elevated levels of circulating triglycerides, lipoproteins, and increased lipolysis, potentially increasing the risk of cardiovascular disease [102–104]. Challenges, such as limited food variety and maintaining long-term adherence, present additional considerations for young patients with obesity. Although ketogenic diets offer potential benefits, the associated risks, such as nutrient deficiencies, growth and developmental impacts, and metabolic complications, necessitate careful monitoring to ensure these diets are applied safely in children [105, 106].

### Fasting-based interventions

Eating pattern-based dietary interventions, including intermittent fasting, time-restricted feeding, and the fasting-mimicking diet, are gaining attention for their potential metabolic benefits. Intermittent fasting (IF) involves alternating cycles of fasting and eating; time-restricted feeding (TRF) restricts daily food intake to a specific time window, typically 6–8 h, promoting a consistent daily fasting period; the fasting-mimicking diet (FMD) entails consuming an extremely low-calorie diet mimicking the physiological effects of fasting, achieving the advantages of fasting without complete food abstention. These approaches are being explored for adaptability and potential health benefits in animal experiments and clinical settings to trigger beneficial metabolic changes that aid weight management and improve overall health by leveraging the body’s natural responses to fasting periods, including improved lipolysis and thermogenesis and glucose management [107–112]. Despite their simplicity and departure from traditional calorie counting, implementing fasting-based strategies in pediatric populations warrants careful evaluation owing to the critical nutritional needs of growing children and adolescents and the potential impact on their physical and cognitive development [113, 114]. Although these fasting methods offer a fresh perspective on dietary management with demonstrated feasibility and positive outcomes [115–118], the evidence supporting their utility as acceptable therapeutic approaches, particularly for younger demographics, is still emerging. Comprehensive research is needed to establish their efficacy and safety and develop age-appropriate guidelines for children and adolescents.

### Dietary supplements

Dietary supplements, including vitamins, nutrients, probiotics, plant extracts, and polyphenols, are increasingly recognized for their potential role in managing childhood obesity [119]. Omega-3 polyunsaturated fatty acids and vitamin D have been extensively researched in pediatric populations. Despite growing interest, their use in children is marked by controversy, largely due to inconsistent clinical outcomes that raise questions regarding treatment efficacy and reliability. For example, some studies have associated omega-3 supplementation with improvements in insulin resistance and fatty liver disease, as well as weight reduction in patients with obesity. In contrast, other studies have suggested no significant effect on body weight, indicating unclear impacts on anthropometric indices [120–122]. This emphasizes the need for larger pediatric studies to ascertain the effectiveness of omega-3.

The focus on probiotics, driven by insights into the role of the human microbiome in health, signals a shift in our understanding of the causes of obesity [123]. Probiotics, specifically *Lactobacillus* and *Bifidobacterium* species, show promise in reducing BMI and improving metabolic parameters, indicating their potential as an intervention for children with metabolic issues. However, cautious use of dietary supplements is recommended owing to limited evidence regarding their safety and effectiveness in children, potential interactions with medications, and unknown long-term health consequences [124–126]. This situation highlights the urgent need for comprehensive clinical trials to verify the safety and benefits of dietary supplement use in childhood obesity treatment.

## Other approaches

Lifestyle interventions are foundational in managing childhood obesity, particularly through increased physical activity and exercise. These approaches are the first line of defense, especially in a preventive and managing manner. Encouraging a healthy diet and regular physical activity are essential, as these modifications can significantly impact overall health and prevent the progression of obesity [127]. Hence, lifestyle interventions are usually combined with pharmacological or dietary interventions to enhance the efficacy of these treatments [1]. This integrative approach is especially crucial when obesity reaches severe levels, as lifestyle changes alone often become insufficient [128–130]. Thus, most clinical treatment approaches for childhood obesity include combined treatment with lifestyle interventions as an effective integrative approach.

Metabolic and bariatric surgery (MBS), including procedures such as sleeve gastrectomy, gastric bypass, and gastric banding, is recognized as the most effective treatment for adolescents with severe obesity, notably reducing appetite and facilitating substantial weight loss alongside improvements in comorbidities and overall quality of life [131, 132]. These surgeries are considered for adolescents under stringent criteria, typically for those with a BMI ≥35 who also have severe comorbidities or a BMI ≥40. Despite the significant benefits, MBS carries potential risks, including nutritional deficiencies, the need for reoperations, and other surgical complications [131]. However, a recent large study indicated that MBS is effective across younger pediatric age groups without affecting vertical growth [133], affirming its utility as a crucial intervention in severe cases of childhood obesity and associated comorbidities. Consequently, this method is increasingly regarded as a viable final option for managing severe childhood obesity, prompting discussions about lowering the stringent criteria for surgery eligibility in younger patients [134].

## Discussion

Managing childhood obesity necessitates a comprehensive approach, incorporating tailored pharmacological and dietary interventions to meet each child’s unique requirements. While appropriate for severe cases, pharmacotherapy must be applied judiciously to prevent adverse impacts on childhood growth and development. Although dietary interventions aiming to alter immediate eating habits and establish long-term nutritional practices for prevention and treatment are perceived as safe, their safety warrants further investigation. The potential synergy between pharmacotherapy and dietary intervention is gaining recognition, showing promise in effectively managing childhood obesity while balancing metabolic control with overall health, as demonstrated in other diseases [129, 135, 136].

The undeniable role of genetics in obesity influences predisposition to the condition and impacts responses to various treatment options. As treatment options progress, obesity management is increasingly likely to prioritize personalized medicine and nutrition, advocating for interventions and dietary plans tailored to each individual’s genetic makeup. The polygenic risk score (PRS) is a tool that estimates an individual’s genetic liability to a trait or disease based on their genotype profile and data from relevant GWAS. Regarding childhood obesity, PRS can be crucial in predicting obesity susceptibility and informing personalized intervention strategies [137]. Several studies have already constructed PRSs specifically for childhood obesity [23, 24, 138, 139], illustrating the potential of genetic insights to guide more effective prevention and treatment approaches.

This personalized approach requires a comprehensive understanding of the genetic factors contributing to obesity and how these interact with various treatment and dietary strategies. It is also essential to refine diagnostic measures to better and, more specifically, diagnose childhood obesity, given the limitations of using BMI as the sole parameter for assessing childhood obesity [140, 141]. Identifying genetic predispositions and tailoring treatments aims to enhance efficacy and minimize adverse effects. Ultimately, this approach would lead to safer and more effective management of childhood obesity, ensuring that interventions are as individualized as the genetic profiles they aim to accommodate.

Future research should explore the molecular mechanisms underlying the interactions among pharmacotherapies, dietary interventions, and genetic factors. In addition to genetic predispositions, understanding the role of gene–environmental interactions is becoming increasingly crucial. Epigenetics—modifications that change gene expression without altering the DNA sequence—mediates the effects of environmental variables on the expression of genes. These modifications include DNA methylation, histone alterations, and microRNA (miRNA) regulation. By affecting how genes are expressed in response to environmental cues, epigenetic mechanisms can contribute to the complexity of obesity pathogenesis and its related metabolic disorders [142–144]. Recognizing these interactions provides valuable insights into how personalized interventions can be tailored to individuals based on genetic makeup, environmental exposures, and lifestyle choices. This integrated approach emphasizes the necessity of advancing our understanding of epigenetics to develop more precise and effective strategies for preventing and managing childhood obesity. Advancements in computational technologies, such as artificial intelligence and high-throughput genomic analysis, promise increased accessibility to personalized treatments in the near future, marking a major step toward more effectively addressing childhood obesity.

## Abbreviations

BMI, body mass index; GWAS, genome-wide association studies; FTO, fat mass and obesity-associated protein; IRX3/5, Iroquois homeobox 3 and 5; SIM1, single-minded family BHLH transcription factor 1; POMC, pro-opiomelanocortin; PCSK1, proprotein convertase subtilisin/Kexin type 1; LEPR, leptin receptor; AGRP, agouti-related protein; MC4R, melanocortin 4 receptor; SH2B1, SH2B adaptor protein 1; MRAP2, melanocortin 2 receptor accessory protein 2; PHIP, Pleckstrin homology domain-interacting protein; FDA, U.S. Food and Drug Administration; GLP1RAs, glucagon-like peptide-1 receptor agonists; GABA, γ-aminobutyric acid; GIPR, glucose-dependent insulinotropic polypeptide receptor; IF, intermittent fasting; TRF, time-restricted feeding; FMD, fasting-mimicking diet; MBS, metabolic and bariatric surgery.
